# The Role of Nutrition in Periodontal Health: An Update

**DOI:** 10.3390/nu8090530

**Published:** 2016-08-30

**Authors:** Shariq Najeeb, Muhammad Sohail Zafar, Zohaib Khurshid, Sana Zohaib, Khalid Almas

**Affiliations:** 1 Restorative Dental Sciences, Al-Farabi College, Riyadh 361724, Saudi Arabia; shariqnajeeb@gmail.com; 2 Department of Restorative Dentistry, College of Dentistry, Taibah University, Al Madinah, Al Munawwarah 41311, Saudi Arabia; 3 College of Dentistry, King Faisal University, P.O. Box 380, Al-Hofuf, Al-Ahsa 31982, Saudi Arabia; drzohaibkhurshid@gmail.com; 4 Department of Biomedical Engineering, School of Engineering, King Faisal University, P.O. Box 380, Al-Hofuf, Al-Ahsa 31982, Saudi Arabia; szohaib@kfu.edu.sa; 5 Division of Periodontology, University of Dammam, College of Dentistry, Dammam 31441, Saudi Arabia; Khalidalmas9@gmail.com

**Keywords:** periodontium, periodontal health, micronutrients, wound healing, periodontal regeneration

## Abstract

Periodontal health is influenced by a number of factors such as oral hygiene, genetic and epigenetic factors, systemic health, and nutrition. Many studies have observed that a balanced diet has an essential role in maintaining periodontal health. Additionally, the influences of nutritional supplements and dietary components have been known to affect healing after periodontal surgery. Studies have attempted to find a correlation between tooth loss, periodontal health, and nutrition. Moreover, bone formation and periodontal regeneration are also affected by numerous vitamins, minerals, and trace elements. The aim of this review is to critically appraise the currently available data on diet and maintenance of periodontal health and periodontal healing. The effects of nutritional intervention studies to improve the quality of life and well-being of patients with periodontal disease have been discussed.

## 1. Introduction

Periodontitis is defined as “an inflammatory disease of the supportive tissues of the teeth caused by specific microorganisms or groups of specific microorganisms, resulting in progressive destruction of the periodontal ligament and alveolar bone with increased probing depth formation, recession, or both” [1]. If untreated, it leads to the progressive loss of the alveolar bone and loss of teeth [2,3]. It has been estimated that 40%–90% of the global population is affected by periodontitis, making it one of the most prevalent epidemics in the world [4,5]. The major microorganisms implicated in periodontal diseases include the red-complex bacteria *Porphyromonas gingivalis*, *Prevotella intermedia*, *Tannerella forsythia*, and *Treponema denticola* [6,7,8]. Additionally, *Fusobacterium nucleatum*, *Prevotella* species, *Eikenella corrodens*, *Peptostreptococcus micros*, and *Campylobacter rectus* have also been found in periodontal pockets [9]. It has been suggested that an aggressive immune response leading to the release of inflammatory cytokines like IL-1β against the microorganisms causes the destruction of the periodontal tissues [10]. A variety of factors have been linked with the etiology of periodontitits [11,12]. The primary cause is poor oral hygiene, which leads to the formation of dental plaque containing microorganisms [13]. In addition to these local elements, a number of systemic factors such as diabetes [5], cardiovascular disease [14,15], and pregnancy [16] have also been associated with periodontitis. Additionally, periodontitis has been suggested to contribute towards instigating the aforementioned systemic diseases [17] and may even trigger adverse pregnancy outcomes [18]. Moreover, habits such as smoking, tobacco, and betel nut chewing and drug abuse also contribute to the progression of periodontitis [19,20].

**Figure 1:**
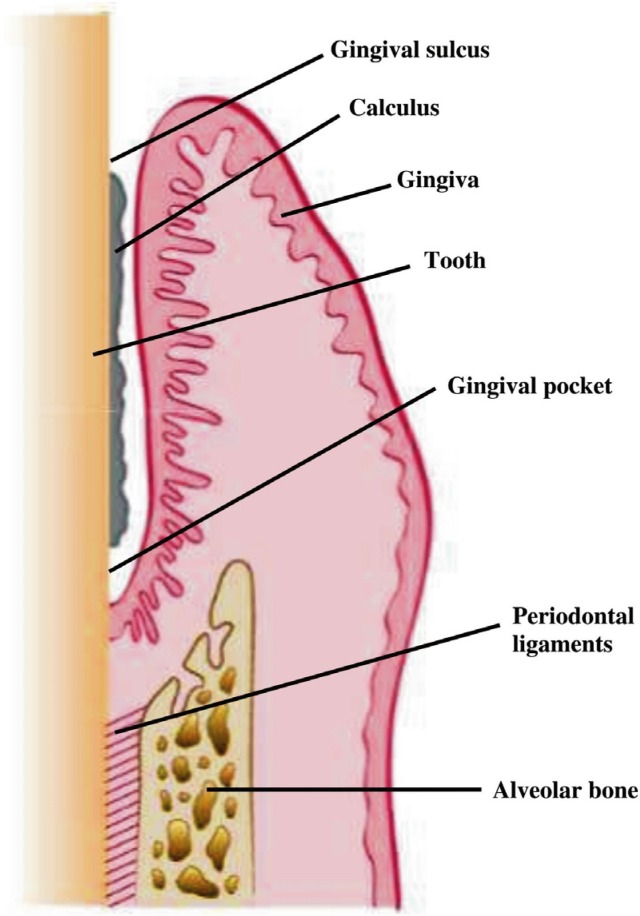
Periodontitis is mainly caused by the presence of calculus, the calcified form of dental plaque. Calculus harbors a number of pathogenic microbes that cause periodontitis. If not treated, periodontal inflammation may cause progressive destruction of periodontal tissues.

Management of periodontitis is achieved mainly by removing the causative factors (dental plaque, microbial biofilm, and calculus) by means of scaling and root planing along with oral hygiene instructions [21,22]. The relationship of calculus, gingival pocket, and periodontal tissues is shown in Figure 1. Antibiotics are considered helpful in severe conditions or in the case of systemic involvement [23]. In order to enhance the functional life of natural teeth with severe periodontal bone loss, guided tissue regeneration (GTR) and bone grafting are employed [24,25]. Implant therapy is used to replace teeth lost due to extensive and uncontrolled degeneration of bone and periodontal tissues [26,27].

Destruction of periodontal tissues is brought about by the production of inflammatory factors released from immune cells. Polymorphonuclear leukocytes are attracted to the site of periodontitis due to the presence of bacteria. Following stimulation by bacterial antigens, leukocytes produce reactive oxidative species (ROS), enzymes, and defensins that degrade the pathogens during phagocytosis [28]. Due to the non-selective nature of ROS, healthy tissues are also affected. Hence, ROS destroy healthy cells during inflammation by damaging DNA and stimulating the production of cytokines from macrophages and monocytes [29]. Furthermore, it has been suggested that ROS may play a part in activating osteoclasts, cells responsible for resorbing bone [30]. Diseased periodontal tissues contain a higher amount of ROS than healthy tissues because of oxidative tissues’ destruction [31,32].

A variety of nutrients have a major impact on periodontal health [33,34,35]. Nutrients are of two types: micronutrients and macronutrients. Micronutrients are those components of food that are required in small or trace amounts. The human diet contains a number of antioxidants in the form of micronutrients [36]. Antioxidant micronutrients include vitamin A (carotenoids and β-carotene) [37], vitamin C (ascorbic acid) [38], vitamin E (α-tocopherol) [39], glutathione [40], and melatonin [41,42]. Studies suggest that antioxidants may overcome the ROS-mediated inflammation of periodontal tissues [43,44].

Macronutrients are nutrients required in large quantities, for example minerals, proteins, carbohydrates, and fats in addition to oxygen and water. High carbohydrate intake has been implicated in periodontal disease and dental caries [45]. The relationship of nutrition and oral health is well known [46,47]. For example, a sugary diet encourages plaque formation and leads to the onset or worsening of dental decay in reaction to poor oral hygiene [48]. A strong link between obesity and periodontal diseases has been reported [45,49]. Indeed, a higher body fat content has been associated with increased gingival bleeding in older patients [50]. On the other hand, polyunsaturated fats (such as omega-3s) have been observed to have a positive effect on periodontal health [51]. A study conducted on Japanese young adults also found an association between high body mass index (BMI) and high risk of periodontal disease [52]. Protein depravation studies conducted on rats in the 1950s resulted in the breakdown of periodontal ligaments, degeneration of gingival tissues, and resorption of the alveolar bone [53,54]. A recent study conducted in Denmark suggested an inverse relationship between high protein intake and periodontitis [55].

In addition, B-vitamin supplementation results in higher clinical attachment following flap surgery [56]. Vitamin D deficiency contributes to negative outcomes following periodontal surgery [57]. Animal studies have found a positive correlation between Vitamin D3 supplementation and osseointegration of dental implants [58]. A higher intake of vitamins A, B, C, and E along with omega-3 fatty acids results in improved healing after non-surgical periodontal therapy [33]. A number of reviews highlighting the link between nutrition and periodontal disease have been published in the last few years. Hence, the aim of this review is to update dental practitioners regarding the link between human nutrition and periodontal health. The effects of nutritional intervention studies to improve the quality of life and well-being of patients with periodontal disease have been discussed.

## 2. Role of Key Nutrients in Periodontal Health

### 2.1. Carbohydrates and Periodontal Health

Plaque is a biofilm of glycoproteins, mucin, and bacteria that adheres to surfaces in the oral cavity [59]. If the plaque remains attached to teeth for a couple of days, it mineralizes to form calculus. The porous calculus provides a potential surface for habitation of periodontal pathogens including *Porphyromonas gingivalis*, *Prevotella intermedia*, *Tannerella forsythia*, and *Treponema denticola* [6,7,8]. Sugar intake has long been established as the major contributing factor in plaque formation. It has been observed that sucrose is more cariogenic than fructose and glucose [60]. Sugars contribute to dental caries and periodontal disease because bacteria ferment them and produce acid, leading to the demineralization of the tooth structure. Studies have revealed that lactose (milk sugar) is less cariogenic than other sugars [48]. There is an established direct relationship between dental caries and the amount and frequency of sugar intake observed in earlier studies [61]. Xylitol, a sugar alcohol produced by the hydrogenation of xylose sugar, is an artificial sweetener used as an alternative to conventional sugars [62,63]. It may have an antibacterial effect against periodontal pathogens such as *Porphyromonas gingivalis* and *Aggregatibacter actinomycetemcomitans* [63,64]. Indeed, oral hygiene measures and regular non-surgical debridement both contribute to the improvement of periodontal health. Hence, a reduction of sugar intake, coupled with scaling, root planing, and the use of xylitol- and maltitol-containing gums have the potential to improve the periodontal health of the general population [62].

### 2.2. Vitamins

Vitamins are essential for general heath and normal functioning. Similarly, various vitamins are required for maintaining health oral and periodontal tissues. Nutritional deficiency of vitamins results in oral manifestations such as scurvy and rickets (Table 1). The role of various vitamins in relation to periodontal health has been discussed. 

#### 2.2.1. Vitamin A

Vitamin A is a fat-soluble vitamin that plays a role in maintaining the integrity of epithelial cells [65]. Dietary sources of vitamin A include eggs, cod liver oil, carrots, capsicum, liver, sweet potato, broccoli, and leafy vegetables. A healthy individual needs approximately 900 µg/day. Deficiency results in retinal disorders (such as night blindness and hyperkerotosis). Considering the antioxidant potential, vitamin A has been used to supplement periodontal treatment [33,56]. 

#### 2.2.2. Vitamin B Complex

The vitamin B complex family consists of vitamins B_1_ (thiamine), B_2_ (riboflavin), B_3_ (niacin), B_5_ (pantothenic acid), B_6_ (pyridoxine, pyridoxal, pyridoxamine) B_7_ (biotin), B_9_ (folic acid), and B_12_ (cobalamins). B vitamins play a vital role in cell metabolism, repair, and proliferation. Deficiency of B vitamins results in a number of diseases and symptoms (Table 1).

Signs and symptoms of B-vitamins deficiency range from dermatitis and paresthesia to oral manifestation such as angular chilitis and glossitis. In addition to anemia, a deficiency of vitamin B_12_ may lead to gingival bleeding. A recent study by Zong et al. found an inverse association between serum vitamin B_12_ levels and the severity of periodontitis [74]. Indeed, previous studies have also found a possible link between low serum vitamin B_12_levels and periodontitis [75]. Additionally, reduced serum vitamin B_9_ levels have been observed in smokers, which may lead to periodontitis [76,77]. However, the mechanism of this association is not clear. In a clinical trial conducted on 30 individuals by Neiva et al., it was observed that B-vitamin complex supplementation accelerates the healing of wounds after periodontal flap surgery [56]. However, more studies are needed to analyze the effect of B vitamins complex supplementation and local application on periodontal health and healing.

#### 2.2.3. Vitamin C

Vitamin C (ascorbic acid) is primarily required for the synthesis of collagen and it also prevents oxidative damage by acting as a ROS scavenger [78]. Scurvy, first identified by Sir Thomas Barlow in 1883, is the name given to the disease caused by the deficiency of vitamin C [79]. In addition to malaise, lethargy, and spots on the skin, periodontal hallmarks of scurvy are bleeding, inflamed, and painful gums. Vitamin C supplementation cures and prevents scurvy [80]. An in vitro study suggests that local application of vitamin C-containing magnesium salt not only improves collagen synthesis but may also decrease ROS-induced inflammation of gingival fibroblasts [81]. Indeed, a dentifrice containing vitamin C-containing magnesium salt has successfully been used to reduce gingival inflammation in a clinical trial by Shimabukuro et al. [82]. Additionally, the vitamin C-containing dentifrice exhibited a significantly higher anti-ROS activity compared to conventional dentifrice. 

Due to its positive effects on periodontal health, vitamin C may also be used in coatings and/or gel forms to enhance the osseointegration of dental implants and improve post-surgical periodontal healing. Ascorbate compounds are potent for scavenging free radicals [35] and help smokers to diminish the breakdown of periodontal tissues by its antioxidant action [83]. The antioxidative properties of vitamin C are discussed later. 

#### 2.2.4. Vitamin D

Vitamin D is required for a number of essential functions in the body. It enhances the absorption of minerals including calcium, magnesium, iron, phosphate, and zinc in the intestine. In humans, there are two important groups of vitamin D, vitamins D_2_ (cholecalciferol) and D_3_ (ergocalciferol). Clinical studies have suggested that a deficiency of dietary vitamin D leads to periodontal inflammation and a delay in post-surgical periodontal healing [34,57,67]. However, other clinical trials have found no significant link between serum vitamin D levels and periodontal health [84,85]. Nevertheless, when the correlation between serum vitamin D levels and disease progression was studied in individuals over 60 years of age, an inverse relationship was observed [86]. Hence, to date, the link between vitamin D deficiency and periodontal health in adults remains unclear and more studies are required to investigate its role. The local effects of vitamin D supplementation on periodontal tissues are more apparent than systemic administration. For example, vitamin D_3_ coated on dental implants may enhance osseointegration with alveolar bone [87]. Furthermore, intraperitoneal injections of vitamin D_3_ accelerates orthodontic tooth movement, making it possible to induce orthodontic tooth movement in patients undergoing bisphosphonate therapy [88,89]. In a clinical trial, systemic supplementation of vitamin D_3_ has not resulted in any added benefits in periodontal bone formation among patients who had undergone maxillary sinus augmentation [90]. Hence, more studies are required to find an association between the outcomes of surgical and non-surgical periodontal therapy with systemic vitamin D_3_ intake.

#### 2.2.5. Vitamin E

Vitamin E (tocopherol) is a fat-soluble vitamin that is considered one of the key extracellular antioxidants. Diets rich in vitamin E include poultry, meat, fish, nuts, seeds, and cereals [68]. Vitamin E stabilizes the membrane structure by terminating the free radical reaction [39,91]. There were no remarkable differences in the plasma level of vitamin E in healthy individuals compared to patients with periodontitis [92]. In contrast, a few studies reported favorable effects of vitamin E in maintaining periodontal health and controlling inflammation. In addition, a reduction of vitamin E was observed in patients with periodontal diseases compared to healthy individuals [93,94]. The level of vitamin E in the alveolar fluid of smokers is reduced, corresponding to increased production of oxidants during smoking [95]. The mechanism of action of vitamin E for periodontal health is not very well understood and needs further research. 

#### 2.2.6. Vitamin K

Vitamin K is a group of vitamins required for the synthesis of proteins that are precursors or prerequisites of the formation of blood coagulation factors such as prothrombin and factors VII, IX, and X [71]. In addition, research has indicated that vitamin K also plays a role in the formation of proteins required for bone metabolism such as osteocalcin and periostin [71,72]. A number of foods such as kale, spinach, collards, and mustard are a source of vitamin K_1_. However, vitamin K_2_ deficiency is rare owing to the fact that anaerobic bacteria present in the gut can convert vitamin K_1_ to K_2_. Nevertheless, intake of antibiotics may disrupt the balance of intestinal bacteria, leading to a deficiency of vitamin K [96]. Hence, if antibiotics are to be made part of periodontal therapy for a long period of time, coagulopathies might be observed. Vitamin K is an important pharmacological agent used to reverse the anticoagulant effects of warfarin and routinely administered for patients undergoing hemodialysis [97]. Hence, if periodontal therapy is to be administered to patients with kidney failure, vitamin K can be used to treat any bleeding incidents. The routine diet provides enough vitamin K for a healthy individual, hence the deficiency of vitamin is very rare. However, a patient with digestive disorder or who has been using antibiotics for a long period of time must be investigated for vitamin K deficiency [98]. Although vitamin K deficiency may lead to gingival bleeding, a recent study by Aral et al. has found that vitamin K supplementation was not able to reduce pro-inflammatory factors in the periodontium [73].

## 3. Antioxidants and Periodontal Health

**Figure 2:**
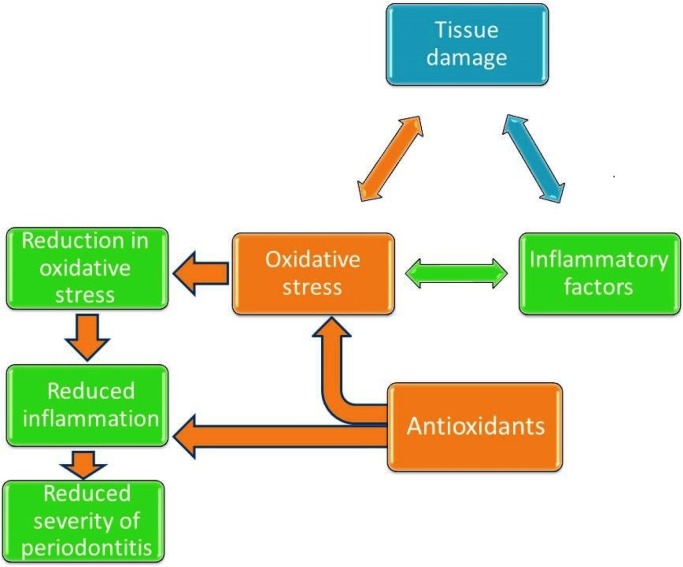
Periodontal disease is a complex process of infective and inflammatory processes leading to production of reactive oxidative species (ROS), which in turn worsen periodontitis. Antioxidants may improve periodontal health and outcomes of periodontal therapy by reducing the oxidative stress via scavenging ROS.

The oral cavity, like any other tissue, undergoes inflammation and injury due to disease and trauma. The inflammatory processes involve the production of ROS by immune cells while stimulated by pathogens [99]. ROS and highly reactive free radicals (molecules containing unpaired electrons) are capable of inflicting cellular and tissue damage by altering the chemical structure of molecules. They particularly damage lipids by initiating a chain of lipid peroxidation [100]. Normally, aerobic respiration leads to the production of ROS. However, the antioxidant defense enzymes reduce the ROS to minimize cellular damage. On the other hand, if there is excessive production of ROS due to inflammation or tissue damage, the antioxidant system is insufficient to minimize oxidative damage. When this balance of ROS production and antioxidant enzymes (e.g., glutathione) is disrupted, a state of oxidative stress occurs [101]. The periodontium can also enter a state of oxidative stress due to the onset of inflammation caused by disease and/or trauma [32]. The association between oxidative stress, inflammation, and inflammatory growth factor is shown in Figure 2. Antioxidants may help in reducing the severity of disease by scavenging ROS. A number of dietary components that can function as antioxidants have shown potential for improving periodontal health and healing.

### 3.1. Vitamins as Antioxidants

Apart from playing a vital role in cell metabolism, vitamins have potent antioxidant properties. Vitamins A, C, and E have all been observed to modulate the anti-oxidant defense system. To date, dietary vitamins C and E have been studied for their potential role in reducing oxidative stress in the periodontium. Supplementation with vitamin C in patients undergoing non-surgical periodontal therapy increased the total antioxidant capacity (TAOC); however, no significant benefit of doing so was found in terms of improving the outcomes of periodontal therapy [66]. On the other hand, in a recent study, increased intake of foods high in vitamins A, C, and E has been observed to decrease the severity of periodontitis in non-smokers; however, the same effects could not be replicated in smokers [33]. Similarly, supplementation with vitamin E has been observed to simultaneously reduce levels of serum superoxide dismutase and improve the outcomes of scaling and root-planing [102]. Indeed, more long-term, well-designed studies are required to ascertain the antioxidant effect of vitamin supplementation on periodontal and peri-implant healing.

### 3.2. Lycopene

Lycopene is a red pigment present in vegetables such as tomatoes, carrots, and watermelons. Lycopene may prevent cancer and cardiac diseases due to its antioxidant effects [103]. Similarly, in some studies lycopene has been investigated as an adjunct to non-surgical periodontal therapy. A study by Chandra et al. suggested that lycopene supplementation may enhance the improvement of periodontal health [104]. Similarly, later studies have also found a possible therapeutic role of lycopene in the management of periodontitis [105,106]. To date, the exact mechanism of action of lycopene in the periodontium has not been established. Indeed, the antioxidative effect of lycopene on the periodontium warrants more research before it may be advocated as a dietary supplement as an adjunct to surgical and non-surgical periodontal therapy. 

### 3.3. Melatonin

Melatonin is a potent antioxidant secreted by various organs of the human body [107]. Additionally, plants and cereals are sources of melatonin. Although melatonin is not classified as a major nutrient, it has been suggested that, in supplement form, the antioxidative properties of melatonin are more potent than those of Vitamin E [108]. More recently, research has focused on the possible therapeutic potential of melatonin supplementation in the oral cavity and, particularly, on the periodontium [109]. Topical forms of melatonin may be used as an adjunct to surgical and non-surgical periodontal therapy. Indeed, studies conducted on animals have shown that melatonin reduces bone resorption caused by induced periodontitis [110]. Furthermore, the anti-ROS effects of melatonin have also led to a reduction in periodontal inflammation and bone loss in diabetic animal models, indicating a potential of melatonin in managing diabetic-induced periodontitis [111]. Clinical studies also suggest that melatonin may have a positive effect on periodontal health [112,113]. Apart from the therapeutic effect of topical melatonin supplementation on periodontal inflammation, research has also focused on its use as an osseoconductive agent around dental implants [114]. Melatonin acts as an ROS scavenger at the site of implant placement to reduce inflammation and stimulates the proliferation of osteoblasts [115]. Animal studies strongly indicate that melatonin gel application at the site of implant placement may promote closer bone-implant contact [116,117,118]. Similar effects of melatonin are seen when applied as a gel on post-extraction sockets [119]. Although melatonin has been approved by the FDA as a dietary supplement for treating sleep disorders, the effect of systemic melatonin on periodontal health and post-surgical healing has not been researched to date. Therefore, future research should focus on the effect of dietary melatonin supplementation on non-surgical and surgical periodontal therapy.

## 4. Dietary Minerals and Trace Elements

Mineral nutrients are elements (other than oxygen, carbon, nitrogen, and hydrogen) required by organisms to survive and function normally. For example, calcium, phosphorus, potassium, sulfur, sodium, chlorine, and magnesium are normally required in abundance. Iron, cobalt, copper, zinc, manganese, molybdenum, iodine, bromine, and selenium are required in small concentrations and, hence, are termed trace minerals. Deficiency of minerals has implications on periodontal health as well [120]. The association between mineral intake and periodontal health is discussed below (Table 2).

### 4.1. Calcium

Calcium is required for the normal functioning of muscles and body systems. Additionally, calcium is essential for the maintenance and formation of calcified tissues such as bone and teeth. Furthermore, it is also required for blood cells to function. Dietary sources of calcium are dairy products, leafy vegetables, nuts, and seeds. A lack of calcium (hypocalcemia) may lead to cardiac arrhythmias, conclusions, tetany, and numbness and/or tingling in hands, feet and round the lips. A deficiency of dietary calcium may impact periodontal health as well. Co-supplementation of calcium and vitamin D is commonly used and has a positive effect on outcomes of periodontal therapy [121]. A study conducted on older Danish patients indicated that a higher intake of dairy products decreases the severity of periodontitis in later life [122]. Similarly, a recent study also suggested an inverse relationship between calcium intake and periodontitis among Danish adults [55]. It has been established that local delivery of calcium, in the form of hydroxyapatite, enhances the osseointegration of dental implants [123]. However, the literature concerning the effect of dietary and supplemental calcium on surgical periodontal therapy and osseointegration of dental implants is scare.

### 4.2. Magnesium

Magnesium is required for cell metabolism and maintenance and formation of bone. The deficiency of magnesium interfered with the parathyroid hormone and directly affects the bone resulting in osteoporosis [128]. Magnesium supplements have been shown to reduce incidences of fractures in osteoporotic patients, indicating their positive effect on the maintenance of bones [129,130]. However, clinicians have to be very careful while prescribing magnesium supplementation as overdose also affects bone health and may increase bone mineral density [128,131]. However, the impact of dietary magnesium on periodontal health is still unclear. So far, only one study has suggested a positive effect of magnesium-rich diets on non-surgical periodontal therapy [124]. Therefore, more research is indeed required to ascertain the effect of dietary magnesium on periodontal health.

### 4.3. Iron

Iron is mainly required for synthesis of proteins, including hemoglobin and enzymes. Foods such as red meat, spinach, fish (tuna and salmon), and beans are rich sources of iron. Iron deficiency leads to anemia and related symptoms. Oral manifestations of anemia include recurrent ulceration, pale mucosa, and burning of the mouth. Indeed, a study indicates that iron-deficiency anemia leads to a reduction in antioxidant enzymes, leading to an increased oxidative stress and worsening of periodontal diseases [125]. Nevertheless, no studies have attempted to find a correlation between iron supplementation and the outcomes of periodontal therapy.

### 4.4. Zinc

Protein-rich foods are the primary source of dietary zinc [132]. Zinc is second to iron as the most abundantly found trace mineral in the human body [133]. Zinc acts as a cofactor in many enzyme-controlled processes. Particularly, it modulates the processes of auto-debridement and keratinocyte migration during wound repair [134]. Furthermore, it also exerts an antioxidative effect by scavenging ROS in addition to neutralizing bacterial toxins [135]. Hence, zinc is an important component of periodontal dressings [136]. Dietary zinc may also play an important role in maintaining periodontal health. It has been suggested that a lack of dietary zinc leads to worsening of periodontal disease in patients with type 2 diabetes mellitus [137]. Indeed a systematic review by Pushparani has further supported the importance of zinc in preventing diabetes-related periodontitis by exerting an anti-oxidant effect [126]. Therefore, zinc supplementation may have the potential to augment the therapeutic effects of periodontal therapy [138]. Although enhancement of osseointegration has been reported around zinc-coated dental implants in osteoporotic rats [139], the effect of reduced serum zinc levels or zinc supplementation on surgical periodontal therapy and osseointegration of dental implants has not been investigated. Indeed, the impact of zinc on wound healing suggests that zinc supplementation may have a positive effect on healing and osseointegration around dental implants.

### 4.5. Fluoride

The anti-caries effects of fluoride have been established for a long time [127]. Fluorine prevents caries by strengthening enamel and cementum due to the formation of fluoroapatite and exerting an antibacterial effect via inhibition of bacterial growth and adhesion [140]. Hence, topical fluoride, in the form of dentifrices, gels, foams, and varnishes has been used as a preventative measure against dental caries [141]. Considering its beneficial roles, fluoride has been incorporated into various restorative materials such as glass ionomers [142,143]. These materials act as reservoirs that are capable of releasing fluoride into the oral cavity and recharging while fluoride is available from toothpaste, mouthwashes, or fluoride-rich foods. Systemic administration of fluoride may be via water, milk, and capsules [141]. However, the American Dental Association (ADA) recommends that systemic supplementation of fluoride should be limited to children who are at a high risk of developing dental caries [127]. Fluoride supplementation of 0.25 to 1 mg per day is recommended depending up on the ppm F already present in drinking water [144]. Additionally, fluoride supplementation may also reduce root resorption caused by orthodontic movement of teeth [145]. Green tea is another nutritional source that is rich in various elements required for oral health including calcium, phosphorus, and fluoride [146].

## 5. Nutrients, Periodontal Health, and Specific Conditions

It is evident from the above discussion that the reduced level of certain nutrients (particularly micronutrients) compromises the periodontal health. A number of factors are involved in reducing the serum level of micronutrients such as genetic or gastrointestinal disorders (affects absorption and bioavailability), poor diet, or lifestyle [147]. Certain physiological changes such as pregnancy and aging may affect the daily requirement of various nutrients. 

### 5.1. Pregnancy

Pregnancy is a physiological condition that results in a number of transitory changes in female body organs including the oral cavity. There are changes in the physiology of almost all body systems including cardiovascular, respiratory, and endocrinal. The physiological changes in the gastrointestinal system (nausea, vomiting, and heartburn) are most relevant to nutritional health [148]. These symptoms may result in loss of appetite, altered diet pattern, or loss of ingested food through vomiting. In terms of periodontal health, pregnant women are more prone to periodontitis, gingivitis, and gingival hyperplasia. Although not very well understood, the increased secretion of estrogen has been linked to periodontal diseases during pregnancy [149]. 

As pregnancy needs special consideration, proper diet and nutritional advice can be vital for the prevention and management of periodontal conditions. As discussed earlier, the beneficial role of using antioxidants for periodontal diseases is well documented. Natural produce (such as broccoli, berries, kiwi, beans, and spinach) is a rich source of antioxidants [147], and pregnant women should be encouraged to consume these foods. In addition, increasing fiber and reducing refined sugar can be advised as preventive measures [147]. Patients should be educated in oral hygiene maintenance as better oral hygiene potentially reduces the amount of oxidants. In case of complications or malnutrition problems, specialist advice or referral to a nutritionist must be considered. 

### 5.2. Ageing Population and Role of Diet Nutrition

Recent advancements in healthcare and quality of life have resulted in a significant increase in life expectancy and average age. In the USA, an average life has increased by 30 years during the last century [150] and is estimated to increase to 100 years by 2050 [151]. Besides average age, the number of centenarians is increasing [152]. For instance, centenarians in Mexican populations numbered under 3000 in 1930 but increased to 19,000 in 2000 and are expected to be more than 135,000 by 2050 [151]. According to an adult dental health survey, a majority of patients (85%) reporting periodontitis were 65 years or older [153]. Numbers of natural teeth are reduced in older age [154], which exposes remaining teeth to higher masticatory stresses. This evidence suggests that the number of older people requiring periodontal treatment is likely to increase in the future. 

Oral health care may be compromised by a number of aging factors such as loss of teeth, oral prosthesis, lack of appetite and masticatory ability, altered taste, and other gastrointestinal conditions [155,156]. In addition, polypharmacy and reduced body metabolism results in the impairment of nutritional status. Medical conditions or medications interfering the metabolism or absorption of nutrients may result in nutritional deficiency. For instance, the risk of folic acid deficiency and related complications has been associated with the elderly population in the USA [157]. Older people are more prone to nutritional deficiencies due to individual factors such as reduced masticatory efficiency and choice of food. The masticatory efficiency is compromised due to the presence of dentures and implants, the lack of natural teeth, and xerostomia [158,159]. Patients’ masticatory efficiency and choices should be considered while discussing nutritional options. For instance, patients may not be able to eat certain types of foods because they are difficult to chew or swallow (beef), hard or crunchy (carrots, crusty bread) or abrasive (potato chips) [160]. In order to manage such issues, a variety of food choices can be offered to patients along with assurance. Considering the role of nutrition for oral and periodontal health, nutritional advice can be very helpful for the prevention and management of periodontal diseases. Regular and timely nutrition consultation during dental practice can improve the quality of life, hence benefitting the elderly [161,162]. In the case of a deficiency of any micronutrients, dietary sources or nutritional supplements must be considered. 

## 6. Conclusions

Although some studies suggest that improving nutrition and supplementation of vitamins and minerals, particularly vitamin C, may contribute to improvement of periodontal health, there are a number of limitations of the current research that should be overcome. The reported treatment effects are too small to indicate the magnitude of therapeutic supplements when used as an adjunct to periodontal therapy. Hence, well-designed, long-term studies are needed to ascertain the direct effects of dietary supplements on the outcomes on periodontal diseases. 

## Figures and Tables

**Table 1:** Major vitamins, their dietary sources, daily requirements, deficiency diseases, and reported importance in periodontal health.

Nutrient	Dietary Source(s)	Importance in Periodontal Heath	Reported Improvement in PD and CAL (Mean mm, SD)	References
Vitamin A	Cod liver oil, carrots, capsicum, liver, sweet potato, broccoli, leafy vegetables	Not clear. Research indicates insignificant improvement in periodontal health upon supplementation.	PD: 0.52 ± 0.03 CAL: n.d.	[33,65]
B-vitamins	B_1_—Liver, oats, pork, potatoes, eggs B_2_—Bananas, dairy, green beans B_3_—Eggs, fish, meat, mushrooms, nuts B_5_—Avocados, meat, broccoli B_6_—Meat, vegetables, nuts, banana B_7_—Raw egg, liver, leafy vegetables, peanuts B_9_—Cereals, leafy vegetables B_12_—Animal products	Supplementation may accelerate post-surgical healing.	PD: 1.57 ± 0.34 CAL: 0.41 ± 0.12	[56]
Vitamin C	Citrus fruits, vegetables, liver	Gingival bleeding and inflammation are hallmarks of scurvy. Supplementation may improve outcomes of periodontal therapy.	PD: 0.58 ± 0.14 CAL: n.d.	[66]
Vitamin D	Fish eggs, mushrooms, liver, milk	Deficiency may lead to delayed post-surgical healing. Local application may accelerate post-surgical healing/osseointegration	PD: 1.35 (SD n.d.) CAL: 1.4 (SD n.d.)	[34,56,57,67]
Vitamin E	poultry, meat, fish, nuts, seeds and cereals	Impaired gingival wound healing	PD: 0.39 ± 0.18 CAL: n.d.	[33,68,69,70]
Vitamin K	Green vegetables, egg yolk	Deficiency may lead to gingival bleeding. No known effects on periodontal therapy if supplementation used as an adjunct.	n.d.	[71,72,73]

PD (Pocket depth), CAL (Clinical attachment level), n.d. (Not determined), SD (standard deviations).

**Table 2:** Major minerals, their dietary sources, daily requirement, deficiencies, and reported importance in periodontal health.

Nutrient	Dietary Source(s)	Importance in Periodontal Heath	Reported Improvement in PD and CAL (Mean mm)	References
Calcium	Milk products, eggs, canned bony fish, leafy vegetables, nuts, seeds	Required for formation of teeth and bones. Supplementation improves outcomes of non-surgical periodontal therapy. Local application enhances osseointegration.	n.d.	[121,122,123]
Magnesium	Cocoa, soybeans, nuts, spinach, marine vegetables, tomatoes	Required for cell metabolism and bone formation. Supplementation may improve outcomes of non-surgical periodontal therapy.		[124]
Iron	Red meat, tuna, dry beans, spinach	Possible anti-oxidant effect on periodontium.	n.d.	[125]
Zinc	Protein-rich foods, spinach, grains	Possible anti-oxidant effect on periodontium. Reduces severity of diabetes-induced periodontitis	n.d.	[126]
Fluoride	Grape fruits, cocoa, tea, dried fruits and nuts, fluoridated water	Supplementation and topical application prevents dental caries.	n.d.	[127]

PD (Pocket depth), CAL (Clinical attachment level), n.d. (Not determined).
